# Assessment of Machine Learning to Estimate the Individual Treatment Effect of Corticosteroids in Septic Shock

**DOI:** 10.1001/jamanetworkopen.2020.29050

**Published:** 2020-12-10

**Authors:** Romain Pirracchio, Alan Hubbard, Charles L. Sprung, Sylvie Chevret, Djillali Annane

**Affiliations:** 1 Department of Anesthesia and Perioperative Medicine, Zuckerberg San Francisco general Hospital and Trauma Center, University of California, San Francisco; 2 Division of Biostatistics, School of Public Health, University of California, Berkeley; 3 Department of Anesthesiology, Critical Care Medicine and Pain, Hadassah Hebrew University Medical Center, Jerusalem, Israel; 4 Department of Biostatistics and Medical Informatics, Institut national de la santé et de la recherche médicale Unite Mixte de Recherche 1153, Hospital Saint Louis, University of Paris, Paris, France; 5 Saclay and Paris Seine Nord Endeavour to Personalize Interventions for Sepsis, Rapid Recognition of Corticosteroid Resistant or Sensitive Sepsis, Department of Intensive Care, Hôpital Raymond Poincaré Groupes Hospitalo-Universitaires Assistance Publique–Hôpitaux de Paris, Université Paris Saclay, Laboratory of Infection and Inflammation, School of Medicine Simone Veil, University Versailles Saint Quentin, University Paris Saclay, Institut national de la santé et de la recherche médicale, Garches, France

## Abstract

**Importance:**

The survival benefit of corticosteroids in septic shock remains uncertain.

**Objective:**

To estimate the individual treatment effect (ITE) of corticosteroids in adults with septic shock in intensive care units using machine learning and to evaluate the net benefit of corticosteroids when the decision to treat is based on the individual estimated absolute treatment effect.

**Design, Setting, and Participants:**

This cohort study used individual patient data from 4 trials on steroid supplementation in adults with septic shock as a training cohort to model the ITE using an ensemble machine learning approach. Data from a double-blinded, placebo-controlled randomized clinical trial comparing hydrocortisone with placebo were used for external validation. Data analysis was conducted from September 2019 to February 2020.

**Exposures:**

Intravenous hydrocortisone 50 mg dose every 6 hours for 5 to 7 days with or without enteral 50 μg of fludrocortisone daily for 7 days. The control was either the placebo or usual care.

**Main Outcomes and Measures:**

All-cause 90-day mortality.

**Results:**

A total of 2548 participants were included in the development cohort, with median (interquartile range [IQR]) age of 66 (55-76) years and 1656 (65.0%) men. The median (IQR) Simplified Acute Physiology Score (SAPS II) was 55 [42-69], and median (IQR) Sepsis-related Organ Failure Assessment score on day 1 was 11 (9-13). The crude pooled relative risk (RR) of death at 90 days was 0.89 (95% CI, 0.83 to 0.96) in favor of corticosteroids. According to the optimal individual model, the estimated median absolute risk reduction was of 2.90% (95% CI, 2.79% to 3.01%). In the external validation cohort of 75 patients, the area under the curve of the optimal individual model was 0.77 (95% CI, 0.59 to 0.92). For any number willing to treat (NWT; defined as the acceptable number of people to treat to avoid 1 additional outcome considering the risk of harm associated with the treatment) less than 25, the net benefit of treating all patients vs treating nobody was negative. When the NWT was 25, the net benefit was 0.01 for the treat all with hydrocortisone strategy, −0.01 for treat all with hydrocortisone and fludrocortisone strategy, 0.06 for the treat by SAPS II strategy, and 0.31 for the treat by optimal individual model strategy. The net benefit of the SAPS II and the optimal individual model treatment strategies converged to zero for a smaller number willing to treat, but the individual model was consistently superior than model based on the SAPS II score.

**Conclusions and Relevance:**

These findings suggest that an individualized treatment strategy to decide which patient with septic shock to treat with corticosteroids yielded positive net benefit regardless of potential corticosteroid-associated side effects.

## Key Points

**Question:**

Can machine learning–derived estimated individual corticosteroid therapy effect yield better results than treat all or treat no one strategies in adults with septic shock?

**Findings:**

In this cohort study using individual patient data from 2548 patients in 4 multicenter trials, the individual estimation-based treatment strategy always yielded a positive net benefit. Compared with individual estimation-based treatment rule, strategies to treat all patients or to treat no one were associated with a worse outcome.

**Meaning:**

These findings suggest that the decision to treat patients with septic shock with hydrocortisone or hydrocortisone and fludrocortisone should be based on the estimated individual treatment effect as derived from machine learning.

## Teaser

This cohort study examines treatment effects of using a machine learning–derived treatment strategy vs treat all or treat none strategies using data from 4 randomized clinical trials among patients with septic shock.

## Introduction

Sepsis continues to place a burden on the health care system worldwide, accounting for approximately 11 million deaths per year.^1^ Apart from eradicating the infection and restoring cell metabolism with oxygen therapy, fluid replacement, and vasopressors, there is no specific treatment for sepsis.^2^ In septic shock, there is moderate evidence from randomized clinical trials (RCTs) that corticosteroids may improve short-term survival.^3,4^ However, in practice, clinicians remain uncertain about the benefit of corticosteroids at the individual level.

Clinical trials are usually performed to estimate the average treatment effect (ATE). The translation of findings into clinical practice follows commonly a binary model in which if a trial yields positive results, all patients are treated, or in case of negative results, no one is treated. This approach assumes that the treatment effect for every patient will be similar to the ATE observed in the original trial. However, individual response to a treatment may substantially vary according to, for example, patient’s baseline characteristics.^5,6,7^ This source of variability is often referred to as *heterogeneity in treatment effect* (HTE).^8,9,10^ Identifying the sources of treatment response heterogeneity (eg, gene variations in oncology) is central to the development of individualized treatment rules and personalized medicine.^8^ Accordingly, individual response to corticosteroids may depend on a patient’s characteristics.^11,12,13^

In case of HTE, accessing data from RCTs offers the opportunity to train models for the estimation of the individual treatment effect (ITE) for a particular intervention.^5,8,14,15^ An estimation model for the ITE could assist in identifying patients likely to respond to treatment vs those unlikely to respond with the aim of guiding clinician decision-making and improving treatment efficiency.^7,14^

Therefore, the primary objectives of this study were to estimate the ITE of corticosteroids in adults with septic shock in intensive care units (ICUs) using machine learning and to evaluate the net benefit of corticosteroids when the decision to treat is based on the individual estimated absolute treatment effect.

## Methods

This study is based on the analysis of 4 RCTs evaluating the benefit of corticosteroids for treating septic shock.^16,17,18,19^ A fifth study^20^ was used to externally validate the results. As a study exclusively based on the analysis of fully deidentified data, this research was considered nonhuman participants research and deemed exempt from informed consent by the Comité des Protection des Personnes Ile de France III. This study was part of the Rapid Recognition of Corticosteroid Resistant or Sensitive Sepsis (RECORDS) program approved by the Comité des Protection des Personnes Ile de France III. This report follows the Strengthening the Reporting of Observational Studies in Epidemiology (STROBE) and Transparent Reporting of a Multivariable Prediction Model for Individual Prognosis or Diagnosis (TRIPOD) reporting guidelines.

### Data

#### Studies

This cohort study used data from 4 RCTs.^16,17,18^ Study characteristics are presented in eTable 1 in the Supplement. The study by Annane et al^16^ found that in corticotropin nonresponders (ie, patients who did not increase their cortisol by 9 μg/dL or more [to convert to nanomoles per liter, multiply by 27.588] in response to 250 μg corticotropin stimulation test), steroid supplementation improved survival.^16^ In the CORTICUS study,^17^ no effect of hydrocortisone on survival was found, regardless of patients’ response to a corticotrophin test. The COIITSS study^18^ reported a 3% absolute reduction in in-hospital mortality in patients receiving hydrocortisone plus fludrocortisone compared with those receiving hydrocortisone alone. In the CRICS-TRIGGERSEP study,^19^ 90-day all-cause mortality was lower among patients who received hydrocortisone plus fludrocortisone than among those receiving placebo. A fifth study by Arabi et al^20^ was used as an external validation cohort. The study by Arabi et al^20^ was stopped for futility at interim analysis after 75 patients were enrolled; although patients in the hydrocortisone group had a higher rate of shock reversal, hydrocortisone was not associated with a reduction in 90-day mortality.

#### Population

We considered adults with septic shock as defined in individual trials. A summary of the inclusion and exclusion criteria for each individual trial is provided in eTable 2 in the Supplement. Missing data were handled by creating a binary missingness indicator. Since missingness may sometimes occur not at random, the indicators were included in the models to account for potential informative missingness.

#### Interventions

The experimental interventions considered for this analysis were hydrocortisone 50 mg as intravenous bolus every 6 hours for 5 to 7 days with or without tapering and hydrocortisone 50 mg as an intravenous bolus given every 6 hours plus enteral fludrocortisone 50 μg daily, given for 7 days without tapering. The control was either placebo or usual care.

#### Outcomes

We considered 90-day mortality the primary outcome. The secondary outcome was 28-day mortality.

### Statistical Analysis

#### ATE and ITE

We defined the ATE as the difference in 90-day mortality should everyone be treated with corticosteroids vs no one being treated. The ITE was defined as the difference in outcome at the individual level, should this patient receive or not receive the treatment.

The ATE was estimated separately for each study included in the analyses based on machine learning fits of the outcome given baseline factors and treatment using the targeted maximum likelihood estimator (TMLE)^21^ adjusting for study, age, sex, admission category (ie, medical, elective surgery, or emergent surgery), severity of illness scores (measured using Simplified Acute Physiology Score [SAPS II]^22^ and Sepsis-related Organ Failure Assessment [SOFA] score^23^), characteristics of infection (hospital- vs community-acquired), infection site and pathogens, adrenal status (ie, baseline cortisol level and cortisol increment after 250 μg of corticotrophin), arterial lactate level, blood glucose levels, maximal dose of norepinephrine equivalent during the first 24 hours, and initial need for mechanical ventilation. The TMLE is a broad estimation framework for data-adaptive estimation methods that facilitates the construction of asymptotically efficient estimators with desirable finite-sample properties.^24^ The ATE was expressed as a relative risk (RR) and an absolute risk reduction (ARR) with their 95% CIs.

The ITE was estimated using 2 different approaches and expressed as an ARR: the baseline severity of illness model and the optimal individual model. First, as previously proposed,^14^ we assumed that the ITE, *ITE_SAPS II_*, could be estimated as the baseline severity of illness as evaluated based on the SAPS II score^14^ minus the baseline multiplied by the RR:

*ITE_SAPS2_* = *P(Y* = 1|*A* = 0, *W*)(1 – *RR*)

in which *Y* is the outcome, *A* is the binary treatment indicator, *W* is the score indicative of the baseline severity, *RR* is the relative risk of *A = 1* (treatment) vs *A = 0* (control) and *P(Y* = 1|*A* = 0, *W*) is the baseline risk. In this particular case, *W* is the SAPS II^14^ and baseline risk is obtained using the equation

logit*P(Y* = 1|*A* = 0, *SAPS2*) = −7.7631 + 0.0737 × *SAPS II* + 0.9971 × ln(*SAPS II* + 1).

We used empirical RR derived from the original trial results and assumed that treatment effect increases linearly with baseline risk.

Alternatively, because treatment effect may not correlate linearly with baseline severity of illness, we developed an optimal individual model, that is, an estimation model for the probability of dying during the first 90 days following ICU admission using individual data from Annane et al,^16^ CORTICUS,^17^ COIITSS,^18^ and CRICS-TRIGGERSEP.^19^ The optimal individual estimation model is a model for (*P*[*Y =* 1*|A,W*]), that is, it includes patients’ characteristics as well as the treatment actually received (ie, hydrocortisone, hydrocortisone + fludrocortisone, or control). Thus, estimating the ITE based on this model does not require the assumption that treatment effect increases linearly with baseline risk. Specifically, the variables included as factors in the estimation models were treatment received (ie, hydrocortisone, hydrocortisone + fludrocortisone, or control), study, age, sex, admission category (ie, medical, elective surgery, or emergent surgery), severity of illness scores (ie, SAPS II^22^ and SOFA score^23^), characteristics of infection (hospital- vs community-acquired), infection site and pathogens, adrenal status (ie, baseline cortisol level and cortisol increment after 250 μg of corticotrophin), arterial lactate level, blood glucose levels, maximal dose of norepinephrine equivalent during the first 24 hours, and initial need for mechanical ventilation. As an alternative to standard regression approaches, we used an ensemble machine learning algorithm called Super Learner.^25^ Within this algorithm, we used 10-fold cross-validation and the cross-validated performance of the area under the curve (AUC) of the receiver operator curve as the measure of fit to derive the final model. The library of algorithms included in the Super Learner included parametric (ie, logistic regression with and without interaction terms, stepwise regression models based on the Akaike information criterion, and Bayesian generalized linear model) and nonparametric learners (ie, generalized additive models, multivariate adaptive regression splines, gradient boosting, random forest, kernel support vector machine, and support vector machine). The wide range was chosen so that the resulting algorithm could virtually flexibly fit any functional form.

#### Net Benefit

We used the net benefit to quantify the impact of treatment initiation strategies account for both the reduction in the event rate and the risk associated with the treatment.^14,15^

Let *D_i_* be the individualized estimation of treatment effect for patient *i*. Let *T* be the threshold for *D*, such that treatment is initiated in patient *i* if *t* < D_i_ and treatment is avoided if *t* > D_i_. If *t* = D_i_, it is uncertain whether the treatment should be prescribed. Hence, the threshold *T* is used to represent the risk associated with the treatment. The net benefit is defined as^15^

*Net benefit* = *decrease in event rate* – *treatment rate* × *T*

More specifically, it is calculated as:
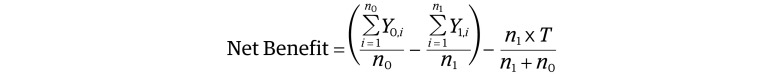
in which *Y_(0,1),i_* is the individual outcome under each treatment option, *n_1_* is the number of patients treated, and *n_0_* is the number of patients not treated.

Based on this definition, the net benefit was calculated as

*Net benefit* = *ATE* – 1 × *T*

for the treat everybody strategy and

*Net benefit* = *ITE_SAPS II_* – *P*[*ITE_SAPS II_* > *T*] × *T*

for the baseline severity of illness strategy, in which *P*[*ITE_SAPS II_*] > *T* is the proportion of patients with an expected reduction in event rate greater than *T* (ie, the treatment rate according to this treatment rule). The optimal individual model strategy was calculated as

*Net benefit* = *ITE_optimal_* – *P*[*ITE_optimal_* > *T*] × *T*.

The net benefit of treating no one serves as the reference and is equal to zero. The net benefit as described by Vickers et al^15^ represents the decrease in the proportion of events associated with treatment minus the proportion of patients treated multiplied by the cost of treatment.

Thus, a negative net benefit means that treating no one is preferable over treating based on a particular strategy (eg, everyone, based on an estimation model, or based on a scoring system) for this particular threshold. In this study, we compare the following treatment strategies: treat all patients, treat no one, treat based on the severity score, or treat based on the Super Learner–derived estimated ITE.

#### Number Willing to Treat

Ideally, the decision threshold takes into account the potential harms secondary to receiving the treatment. For instance, if the harm associated with experiencing the outcome is considered to be 10-fold worse than those of treatment adverse effects, the appropriate decision threshold is 10%. In this case, the treatment should be initiated only in individuals whose estimated absolute treatment effect exceeds 10%. Usually, however, clinicians do not make a decision based on a decision threshold but rather evaluate what is the maximum acceptable number of patients needed to treat to avoid 1 outcome event. In this context, Dorresteijn et al^14^ proposed to use the number willing to treat (NWT), defined as the inverse of the decision threshold. If treating 10 patients is assumed to generate as much harm as 1 outcome event, clinicians would be willing to treat up to 10 patients to prevent 1 event. In such case, the NWT is 10, which is equal to 1 / *T* in which T is 10%.

The possible adverse effects of a short-course corticosteroid administration include superinfection, hyperglycemia, hypernatremia, metabolic alkalosis, gastrointestinal bleeding, psychiatric disturbances, and muscle weakness, with frequency ranging from 1% to 85%.^16,17,18,19^ Therefore, as suggested by Dorresteijn et al,^14^ we calculated the net benefit for a range of possible values of NWT.

#### Performance of the Estimation Models

The performance of the estimation model was evaluated both internally and externally using the data from a different trial.^20^ To evaluate the discrimination performance of the model, we computed the cross-validated AUC together with its 95% CI. Model calibration was evaluated by plotting the estimated probability vs observed prevalence of the outcome and by computing the Brier score.^26^ The same metrics were estimated in the external validation cohort.^20^

#### Decision Trees

To help clinicians decide if a given patient should be receiving steroids or not, we complemented the analysis by generating a decision tree based on age, sex, admission category (ie, medical, elective surgery or emergent surgery), SAPS II, SOFA score, characteristics of infection (ie, hospital- vs community-acquired), infection site, adrenal status (baseline cortisol level and cortisol increment after 250 μg of corticotrophin), arterial lactate level, and maximal dose of norepinephrine equivalent during the first 24 hours. The decision tree was generated using a pruned recursive partitioning algorithm. The complexity parameter was optimized using 20-fold cross validation.

All statistical analyses were performed on R statistical software version 3.5.1 (R Project for Statistical Computing). running on macOS (Apple) platform. *P* values were 2-sided, and statistical significance was set at .05. Data were analyzed from September 2019 to February 2020.

## Results

The training cohort used data from 2548 patients, including 299 patients from Annane et al,^16^ 499 patients from CORTICUS,^17^ 509 patients from COIITSS,^18^ and 1241 patients from CRICS-TRIGGERSEP.^19^ Of these, 515 patients received hydrocortisone alone, 1009 patients received hydrocortisone plus fludrocortisone, and 1024 patients received a placebo or no treatment. Patients characteristics are presented in eTable 3 in the Supplement. Median (interquartile range [IQR]) age was 66 (55-76) years, and 1656 (65.0%) were men. Median (IQR) SAPS II was 55 (42-69), and median (IQR) SOFA score on day 1 was 11 (9-13).

### Individual Studies and Pooled ATE

The Table provides the point estimates for the ATE of corticosteroids on 90-day mortality as reported in individual study as well as the ATE pooled across studies. Compared with the control, corticosteroids (hydrocortisone or hydrocortisone + fludrocortisone) decreased the risk of death at 90 days (RR, 0.89; 95% CI, 0.83 to 0.96; *P* = .004; ARR, 5.11%; 95% CI, 1.50% to 8.72%). Compared with the control, hydrocortisone decreased the risk of dying at 90 days (RR, 0.88; 95% CI, 0.79 to 0.97; *P* = .01; ARR, 6.32%; 95% CI, 1.47% to 11.18%). Similar results were found for the combination hydrocortisone with fludrocortisone (RR, 0.92; 95% CI, 0.85 to 0.99; *P* = .048; ARR, 3.7%; 95% CI, 0.23% to 7.64%). There was no significant difference in the risk of dying at 90 days between hydrocortisone vs hydrocortisone with fludrocortisone (RR, 1.07; 95% CI, 0.96 to 1.19; *P* = .22; ARR, −2.60%; 95% CI, −7.46% to 2.27%). The effects of corticosteroids on 28-day mortality are also presented in the Table.

**Table.** Estimated Treatment Effect on 90-Day and 28-Day Mortality

Outcome	No.	RR (95%CI)	*P* value
**90-d mortality**			
Individual study			
Annane et al^16^^a^	299	0.92 (0.69-1.21)	.54
CORTICUS^17^^b^	499	1.06 (0.81-1.39)	.70
COIITSS^18^^c^	509	0.96 (0.74-1.25)	.76
CRICS-TRIGGERSEP^19^^a^	1241	0.88 (0.78-0.99)	.03
Pooled results			
Any steroid (n = 1524) vs placebo (n = 1024)	2548	0.89 (0.83-0.96)	.01
Hydrocortisone (n = 515) vs placebo (n = 1024)	1539	0.88 (0.79-0.97)	.01
Hydrocortisone + fludrocortisone (n = 1009) vs placebo (n = 1024)	2033	0.92 (0.85-0.99)	.05
Hydrocortisone alone (n = 515) vs hydrocortisone + fludrocortisone (n = 1009)	1524	1.07 (0.96-1.19)	.22
**28-d mortality**			
Individual study			
Annane et al^16^^a^	299	0.54 (0.31-0.97)	.04
CORTICUS^17^^b^	499	1.09 (0.84-1.41)	.51
COIITSS^18^^c^	509	0.96 (0.72-1.27)	.73
CRICS-TRIGGERSEP^19^^a^	1241	0.87 (0.75-1.01)	.06
Pooled results			
Any steroid (n = 1524) vs placebo (n = 1024)	2548	0.89 (0.81-0.97)	.01
Hydrocortisone (n = 515) vs placebo (n = 1024)	1539	0.89 (0.79-1.01)	.08
Hydrocortisone + fludrocortisone (n = 1009) vs placebo (n = 1024)	2033	0.91 (0.82-1.00)	.05
Hydrocortisone alone (n = 515) vs hydrocortisone + fludrocortisone (n = 1009)	1524	1.05 (0.92-1.19)	.46

Abbreviation: RR, relative risk.

^a^ Compares hydrocortisone with fludrocortisone vs placebo.

^b^ Compares hydrocortisone vs placebo.

^c^ Compares hydrocortisone with fludrocortisone vs hydrocortisone.

### Baseline Severity of Illness and Optimal Individual Model

**Figure 1.**
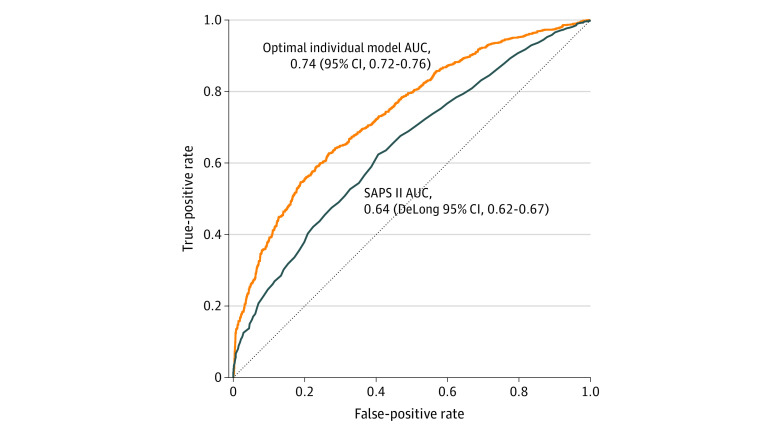
Model Discrimination AUC indicates area under the receiver operating characteristic curve; SAPS II, Simplified Acute Physiology Score.

The observed mortality rate at 90 days was 47.7% (95% CI, 45.7% to 49.6%) (eTable 3 in the Supplement). Based on the SAPS II, the mean estimated probability of death was 55.0% (95% CI, 53.8% to 56.1%)in the overall sample (eTable 4 in the Supplement). The AUC of the SAPS II was 0.64 (DeLong 95% CI, 0.62 to 0.67) (Figure 1).

**Figure 2.**
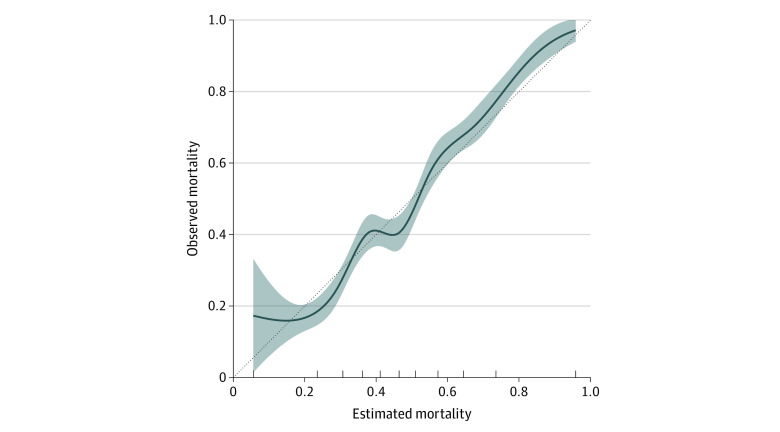
Optimal Individual Model Calibration Calibration plot using 10-fold cross-validation. The dotted line indicates perfect calibration; blue line, calibration obtained with the Super Learner; and shading, 95% CI.

Based on the optimal individual model, the mean estimated probability of death was of 47.7% (95% CI, 46.8% to 47.8%) in the overall sample (eFigure 1 in the Supplement). The optimal individual model discrimination is illustrated in Figure 1. The cross-validated AUC was 0.74 (95% CI, 0.72 to 0.76). Figure 2 illustrates the good calibration of the optimal model (Brier score = 0.21). The estimation performance was similar when using 28-day mortality as the outcome (cross-validated AUC, 0.74; 95% CI, 0.72 to 0.76; Brier score = 0.20). In the external validation cohort, the AUC of the optimal individual model of patients was 0.77 (DeLong 95% CI, 0.59 to 0.92), and the Brier score was 0.28.

The distribution of the ITE for each corticosteroid regimen is illustrated in eFigure 2 in the Supplement . Using the baseline severity of illness model to decide which treatment individual patients should be receiving, the estimated mean ARR was of 5.85% (95% CI, 5.73% to 5.97%) (eFigure 3 in the Supplement). Using the optimal individual model, the estimated mean ARR was of 2.90% (95% CI, 2.79% to 3.01%).

### Net Benefit and NWT

**Figure 3.**
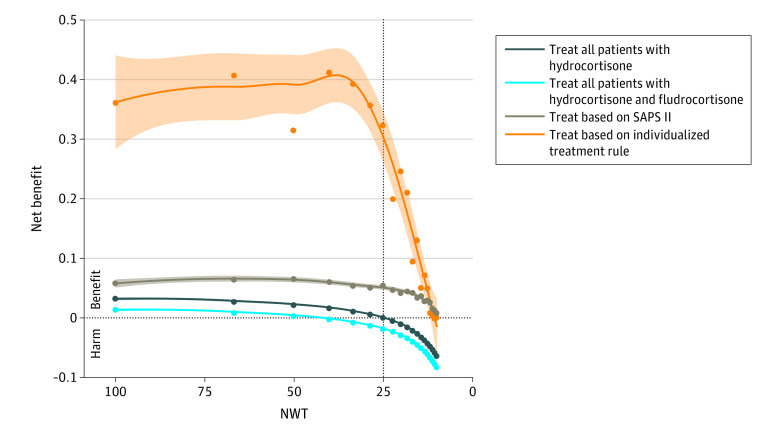
Expected Net Benefit Based on the Number Willing to Treat (NWT) The y-axis is the net benefit for each treatment strategy compared with treating no one. Treating no one served as a reference and is equal to zero. For treat all patients and treat based on the Simplified Acute Physiology Score (SAPS II), the treatment considered is either hydrocortisone alone or hydrocortisone with fludrocortisone. For the optimal individual model, the treatment is the one expected to produce the maximal effect at the individual level. The x-axis is the NWT, which is equal to 1 / decision threshold. Shading indicates 95% CI.

As illustrated in Figure 3, the expected net benefit seemed to highly depend on the treatment strategy. The net benefit of the treat everybody strategy of treating all patients with hydrocortisone or hydrocortisone with fludrocortisone was positive for any NWT greater than 25, meaning that treating all patients with hydrocortisone or hydrocortisone with fludrocortisone was superior to treating no one if the NWT was high (ie, very little harm associated with treatment) but not if the NWT was low (ie, considerable harm associated with treatment). For an NWT of approximately 25, the benefits of treating all patients and treating no one were equivalent (net benefit close to zero). When the NWT decreased to less than 25, the net benefit of treating all patients with hydrocortisone or with hydrocortisone and fludrocortisone was found to be negative, meaning that treating all patients with hydrocortisone or hydrocortisone with fludrocortisone was inferior to treating no one.

Using the estimation-based treatment strategies (ie, based on the severity of illness model or on the optimal individual model) were consistently associated with greater net benefit than treating all patients, regardless of the NWT (Figure 3). While both estimation-based net benefit curves converged to zero for very low NWT values, a treatment strategy based on the optimal individual model was significantly more beneficial than treating based only on the SAPS II. When the NWT was 25, the net benefit was 0.01 for the treat all with hydrocortisone strategy and −0.01 for the treat all with hydrocortisone and fludrocortisone strategy at the cost of treating 100% of patients; the net benefit was 0.06 for the treat by SAPS II strategy at the cost of treating 13.3% of patients 0.31 for the treat by optimal individual model strategy at the cost of treating 14.9% of patients. eFigure 4 in the Supplement illustrates the net benefit according to the proportion of patients who received the treatment for each estimation-based strategy. None of these results were substantially altered when using 28-day mortality as the outcome. The net benefit associated with the optimal individual model in the external validation cohort is illustrated in eFigure 5 in the Supplement.

### Interpretation for Clinical Practice

eTable 4 in the Supplement illustrates the difference in characteristics between the patients with a low estimated ITE (first quartile of the ITE distribution) vs high predicted ITE (last quartile of the ITE distribution). eFigure 6 in the Supplement proposes a decision tree for an NWT of 50, corresponding to a decision threshold of 0.02.

## Discussion

This cohort study found that a personalized approach based on the estimated ITE to decide if a patient with septic shock should be treated with corticosteroids was never harmful to the patients, regardless of potential corticosteroid-related adverse effects. Conversely, a treatment policy based on the ATE (ie, treat all patients or treat no one) identified from RCTs and meta-analyses may generate more harm than benefit at the individual level.

RCTs are usually used to estimate the ATE, which is then interpreted in binary manner, whereby for a positive result, the recommendation is to treat all patients and for a negative result, the recommendation is to treat no one. RCTs are considered as the criterion standard for evidence-based medicine. However, as first reported by Hill et al in 1966,^27^ the ATE is probably not the most informative measure of treatment effect for a clinician who is seeking the best treatment strategy for a particular patient. This is owing to HTE, which describes how treatment’s effect varies across individuals^8^ and can be defined as nonrandom variability in a treatment effect, explaining that the individual response to treatment may vary substantially from the ATE. Different approaches have been proposed to deal with the, including subgroup analysis to identify clusters of patients characterized by a more homogeneous response to treatment.^8^ An alternative and arguably superior approach is to develop accurate, multivariable models to estimate which treatment option is likely to be best at the individual level.^8,14^ This estimation approach can rely on risk modeling, whereby treatment effect is reported across risk strata for the primary outcome. In this study, we used a baseline risk modeling approach based on the SAPS II and showed that a treatment decision based on risk modeling yielded superior net benefit that a decision purely based on the ATE ignoring the heterogeneity among patients in the impact of treatment.

The estimation approach can be more complex and rely on treatment effect modeling.^8^ Although still not commonly adopted in critical care, direct forecasting of the ITE based on patients’ characteristics from RCTs databases has already been applied to cancer^28,29,30^ and cardiovascular research.^31,32,33^ Dorresteijn et al^14^ used the data from RCTs on the benefit of statins to estimate treatment effect for individual patients and showed than the treatment effect modeling approach was associated with more net benefit than treating everyone or no one. Likewise, for corticosteroids in septic shock, we found that a treatment effect modeling approach is superior to a treatment decision strategy based on the ATE and a baseline risk modeling approach. The outcome of treating patients with septic shock with corticosteroids was evaluated based on the net benefit to account for potentially severe adverse effects associated with this class of medication. For each treatment strategy, the net benefit was estimated using a range of adverse effect severity. This range was expressed using the NWT, in which the higher the NWT, the fewer adverse effects associated with the drug and vice versa. Using this approach, we found that a treatment strategy based on the optimal individual model was consistently superior to other approaches. Moreover, for any NWT less than 25, the net benefit of treating all patients with corticosteroids (hydrocortisone or hydrocortisone + fludrocortisone) was found to be negative.

The Super Learner was used to model the probability of death during the first 90 days following admission. This ensemble machine learning approach was shown to be mathematically optimal, that is, have oracle properties.^25^ The Super Learner was previously shown to perform better than a number of alternative modeling approaches in the context of mortality estimation in the ICU.^34^ It was also shown to be a method of choice when dealing with heterogeneity in treatment effect.^35^ Consistently, we found that the Super Learner-based estimation model used to estimate the ITE was associated with better discrimination properties than the SAPS II and more importantly, with excellent calibration. Hence, the net benefit associated with the Super Learner-derived optimal individual model was superior to the treat all strategy and the strategy based on SAPS II. Of note, since the severity of illness model approach relies on multiplying the baseline risk by 1 minus the RR, it does not allow for ITE to go in opposite directions based on patients’ characteristics. This important difference between the 2 approaches is illustrated in eFigure 3 in the Supplement. Finally, the SAPS II is well known to overestimate the probability of death,^34,36^ thereby resulting in an inflation of the estimated net benefit. Interestingly, Luedtke et al^37^ have shown that the Super Learner can be used not only to unbiasedly estimate the ATE but also to learn a treatment rule based on covariates and estimate the impact of using this optimal rule.

### Limitations

This study has some limitations. First, the results may not be generalizable to all patients since the data used to derive the individual estimations are constrained by the inclusion and exclusion criteria used in the RCTs^16,17,18,19^ used to train the models. However, these 4 studies^16,17,18,19^ were selected because they yielded conflicting results regarding the overall benefit of treating patients with steroids, they represent a typical situation in which the estimation of ITE can be used to identify those who will benefit from treatment. To challenge the performance of our algorithm, we tested it using data from an external trial^20^ and, despite a limited size and a substantially higher death rate, found overall good performance. In the future, making the ITE estimation model available to all would be a way to address this limitation by prospectively collecting additional observational data and further recalibrating the models to improve their performance in a particular environment. Second, we used the SAPS II as an alternative to the optimal individual model to identify patients who may benefit from receiving corticosteroids. The SAPS II was developed to estimate hospital mortality, while we used 90-day mortality as the primary outcome measure. Nevertheless, we found consistent results for 28-day mortality and 90-day mortality. The SOFA score^23^ is often preferred by clinicians to evaluate illness severity in the ICU. However, since this score was not intended to estimate mortality, there is no direct way to use it to estimate the ITE. The validation cohort included adults with cirrhosis and septic shock.^20^ While this is a very specific group of patients, it helped to challenge even further the performance of the algorithm. Third, to refine the individualized treatment strategy, one would need to choose the appropriate NWT accounting for the frequency and the severity of adverse effects. Fourth, the decision tree generated to illustrate the use of the ITE in clinical practice should be considered with caution, and the net benefit of such an estimation-based treatment strategy would have to be confirmed prospectively.

## Conclusions

This cohort study found that an individualized estimation-based treatment strategy to decide which patients with septic shock to treat with corticosteroids and which corticosteroid regimen to administer yielded positive net benefit regardless of potential corticosteroid-associated adverse effects. This promising result will need to be validated in a prospective manner.

## Notes

Supplement. **eTable 1.** Description of Studies Included in the Analysis **eTable 2.** Inclusion and Exclusion Criteria for Each Trial **eTable 3.** Characteristics of the Population **eFigure 1.** Estimated Probability of 90-day Mortality Based on SAPS II and on the Optimal Individual Model **eFigure 2.** Distribution of the Estimated Individual Treatment Effect by Steroid Regimen **eFigure 3.** Distribution of Maximal Absolute Risk Difference by Treatment Strategy **eFigure 4.** Net Benefit According to the Proportion of Patients Receiving Treatment **eFigure 5.** Estimated Net Benefit Based Number Willing to Treat in the External Validation Cohort **eFigure 6.** Decision Tree for a Number Willing to Treat of 50 Patients **eTable 4.** Characteristics of the Patients With a Estimated Individual Treatment Effect Within the First vs Fourth Quartiles

## References

[1] Rudd KE, Johnson SC, Agesa KM, Global, regional, and national sepsis incidence and mortality, 1990-2017: analysis for the Global Burden of Disease Study. Lancet. 2020;395(10219):200-211. doi:10.1016/S0140-6736(19)32989-7 31954465 PMC6970225

[2] Rhodes A, Evans LE, Alhazzani W, Surviving Sepsis campaign: international guidelines for management of sepsis and septic shock: 2016. Crit Care Med. 2017;45(3):486-552. doi:10.1097/CCM.0000000000002255 28098591

[3] Annane D, Bellissant E, Bollaert PE, Corticosteroids for treating sepsis in children and adults. Cochrane Database Syst Rev. 2019;12:CD002243. doi:10.1002/14651858.CD002243.pub4 31808551 PMC6953403

[4] Zhang S, Chang W, Xie J, Wu Z, Yang Y, Qiu H The efficacy, safety, and optimal regimen of corticosteroids in sepsis: a Bayesian network meta-analysis. Crit Care Explor. 2020;2(4):e0094. doi:10.1097/CCE.0000000000000094 32426736 PMC7188436

[5] Glasziou PP, Irwig LM An evidence based approach to individualising treatment. BMJ. 1995;311(7016):1356-1359. doi:10.1136/bmj.311.7016.1356 7496291 PMC2551234

[6] Kent DM, Hayward RA Limitations of applying summary results of clinical trials to individual patients: the need for risk stratification. JAMA. 2007;298(10):1209-1212. doi:10.1001/jama.298.10.1209 17848656

[7] Rothwell PM Can overall results of clinical trials be applied to all patients? Lancet. 1995;345(8965):1616-1619. doi:10.1016/S0140-6736(95)90120-5 7783541

[8] Kent DM, Steyerberg E, van Klaveren D Personalized evidence based medicine: predictive approaches to heterogeneous treatment effects. BMJ. 2018;363:k4245. doi:10.1136/bmj.k4245 30530757 PMC6889830

[9] Byar DP Assessing apparent treatment—covariate interactions in randomized clinical trials. Stat Med. 1985;4(3):255-263. doi:10.1002/sim.4780040304 4059716

[10] Iwashyna TJ, Burke JF, Sussman JB, Prescott HC, Hayward RA, Angus DC Implications of heterogeneity of treatment effect for reporting and analysis of randomized trials in critical care. Am J Respir Crit Care Med. 2015;192(9):1045-1051. doi:10.1164/rccm.201411-2125CP 26177009 PMC4642199

[11] Briegel J, Huge V, Möhnle P Hydrocortisone in septic shock: all the questions answered? J Thorac Dis. 2018;10(17)(suppl 17):S1962-S1965. doi:10.21037/jtd.2018.04.120 30023091 PMC6036032

[12] Yende S, Thompson BT Evaluating glucocorticoids for sepsis: time to change course. JAMA. 2016;316(17):1769-1771. doi:10.1001/jama.2016.13904 27695850

[13] Sen A, Yende S Towards personalized medicine in sepsis: quest for Shangri-La? Crit Care. 2013;17(1):303. doi:10.1186/cc12485 23398880 PMC4056785

[14] Dorresteijn JAN, Visseren FLJ, Ridker PM, Estimating treatment effects for individual patients based on the results of randomised clinical trials. BMJ. 2011;343:d5888. doi:10.1136/bmj.d5888 21968126 PMC3184644

[15] Vickers AJ, Kattan MW, Daniel S Method for evaluating prediction models that apply the results of randomized trials to individual patients. Trials. 2007;8:14. doi:10.1186/1745-6215-8-14 17550609 PMC1914366

[16] Annane D, Sébille V, Charpentier C, Effect of treatment with low doses of hydrocortisone and fludrocortisone on mortality in patients with septic shock. JAMA. 2002;288(7):862-871. doi:10.1001/jama.288.7.862 12186604

[17] Sprung CL, Annane D, Keh D, ; CORTICUS Study Group Hydrocortisone therapy for patients with septic shock. N Engl J Med. 2008;358(2):111-124. doi:10.1056/NEJMoa071366 18184957

[18] Annane D, Cariou A, Maxime V, ; COIITSS Study Investigators Corticosteroid treatment and intensive insulin therapy for septic shock in adults: a randomized controlled trial. JAMA. 2010;303(4):341-348. doi:10.1001/jama.2010.2 20103758

[19] Annane D, Renault A, Brun-Buisson C, ; CRICS-TRIGGERSEP Network Hydrocortisone plus fludrocortisone for adults with septic shock. N Engl J Med. 2018;378(9):809-818. doi:10.1056/NEJMoa1705716 29490185

[20] Arabi YM, Aljumah A, Dabbagh O, Low-dose hydrocortisone in patients with cirrhosis and septic shock: a randomized controlled trial. CMAJ. 2010;182(18):1971-1977. doi:10.1503/cmaj.090707 21059778 PMC3001503

[21] van der Laan MJ, Rubin D Targeted maximum likelihood learning. Int J Biostat. 2006;2(1). doi:10.2202/1557-4679.1043 PMC3126670 21969992

[22] Le Gall JR, Lemeshow S, Saulnier F A new Simplified Acute Physiology Score (SAPS II) based on a European/North American multicenter study. JAMA. 1993;270(24):2957-2963. doi:10.1001/jama.1993.03510240069035 8254858

[23] Vincent JL, Moreno R, Takala J, The SOFA (Sepsis-related Organ Failure Assessment) score to describe organ dysfunction/failure: on behalf of the Working Group on Sepsis-Related Problems of the European Society of Intensive Care Medicine. Intensive Care Med. 1996;22(7):707-710. doi:10.1007/BF01709751 8844239

[24] Van der Laan MJ, Rose S Targeted Learning: Causal Inference for Observational and Experimental Data. Springer; 2011. doi:10.1007/978-1-4419-9782-1

[25] van der Laan MJ, Polley EC, Hubbard AE Super Learner. Stat Appl Genet Mol Biol. 2007;6(1):e25. doi:10.2202/1544-6115.1309 17910531

[26] Brier GW Verification of forecasts expressed in terms of probability. Monthly Weather Review. 1950;78(1):1-3. doi:10.1175/1520-0493(1950)078<0001:VOFEIT>2.0.CO;2

[27] Hill AB Reflections on controlled trial. Ann Rheum Dis. 1966;25(2):107-113. doi:10.1136/ard.25.2.107 5326437 PMC2453381

[28] Gill S, Loprinzi CL, Sargent DJ, Pooled analysis of fluorouracil-based adjuvant therapy for stage II and III colon cancer: who benefits and by how much? J Clin Oncol. 2004;22(10):1797-1806. doi:10.1200/JCO.2004.09.059 15067028

[29] Loprinzi CL, Thomé SD Understanding the utility of adjuvant systemic therapy for primary breast cancer. J Clin Oncol. 2001;19(4):972-979. doi:10.1200/JCO.2001.19.4.972 11181659

[30] Steyerberg EW, Homs MYV, Stokvis A, Essink-Bot M-L, Siersema PD; SIREC Study Group Stent placement or brachytherapy for palliation of dysphagia from esophageal cancer: a prognostic model to guide treatment selection. Gastrointest Endosc. 2005;62(3):333-340. doi:10.1016/S0016-5107(05)01587-7 16111947

[31] Rothwell PM, Warlow CP Prediction of benefit from carotid endarterectomy in individual patients: a risk-modelling study: European Carotid Surgery Trialists’ Collaborative Group. Lancet. 1999;353(9170):2105-2110. doi:10.1016/S0140-6736(98)11415-0 10382694

[32] Kent DM, Hayward RA, Griffith JL, An independently derived and validated predictive model for selecting patients with myocardial infarction who are likely to benefit from tissue plasminogen activator compared with streptokinase. Am J Med. 2002;113(2):104-111. doi:10.1016/S0002-9343(02)01160-9 12133748

[33] Califf RM, Woodlief LH, Harrell FEJ Jr, ; GUSTO-I Investigators Selection of thrombolytic therapy for individual patients: development of a clinical model. Am Heart J. 1997;133(6):630-639. doi:10.1016/S0002-8703(97)70164-9 9200390

[34] Pirracchio R, Petersen ML, Carone M, Rigon MR, Chevret S, van der Laan MJ Mortality prediction in intensive care units with the Super ICU Learner Algorithm (SICULA): a population-based study. Lancet Respir Med. 2015;3(1):42-52. doi:10.1016/S2213-2600(14)70239-5 25466337 PMC4321691

[35] Künzel SR, Sekhon JS, Bickel PJ, Yu B Metalearners for estimating heterogeneous treatment effects using machine learning. Proc Natl Acad Sci U S A. 2019;116(10):4156-4165. doi:10.1073/pnas.1804597116 30770453 PMC6410831

[36] Poole D, Rossi C, Latronico N, Rossi G, Finazzi S, Bertolini G; GiViTI Comparison between SAPS II and SAPS 3 in predicting hospital mortality in a cohort of 103 Italian ICUs: is new always better? Intensive Care Med. 2012;38(8):1280-1288. doi:10.1007/s00134-012-2578-0 22584793

[37] Luedtke AR, van der Laan MJ Super-learning of an optimal dynamic treatment rule. Int J Biostat. 2016;12(1):305-332. doi:10.1515/ijb-2015-0052 27227726 PMC6056197

