# Supplementary material for: Assessment of Machine Learning to Estimate the Individual Treatment Effect of Corticosteroids in Septic Shock
Source: JAMA Netw Open. 2020 Dec 10;3(12):e2029050. doi: 10.1001/jamanetworkopen.2020.29050 (PMC7729430; doi:10.1001/jamanetworkopen.2020.29050)
Supplement: Supplement. — eTable 1. Description of Studies Included in the Analysis eTable 2. Inclusion and Exclusion Criteria for Each Trial eTable 3. Characteristics of the Population eFigure 1. Estimated Probability of 90-day Mortality Based on SAPS II and on the Optimal Individual Model eFigure 2. Distribution of the Estimated Individual Treatment Effect by Steroid Regimen eFigure 3. Distribution of Maximal Absolute Risk Difference by Treatment Strategy eFigure 4. Net Benefit According to the Proportion of Patients Receiving Treatment eFigure 5. Estimated Net Benefit Based Number Willing to Treat in the External Validation Cohort eFigure 6. Decision Tree for a Number Willing to Treat of 50 Patients eTable 4. Characteristics of the Patients With a Estimated Individual Treatment Effect Within the First vs Fourth Quartiles [file jamanetwopen-e2029050-s001.pdf]

Supplemental Online Content

Pirracchio R, Hubbard A, Sprung CL, Chevret S, Annane D; for the Rapid Recognition of Corticosteroid Resistant or Sensitive Sepsis (RECORDS) Collaborators. Assessment of machine learning to estimate the individual treatment effect of corticosteroids in septic shock. *JAMA Netw Open*. 2020;3(12):e2029050. doi:10.1001/jamanetworkopen.2020.29050

eTable 1. Description of Studies Included in the Analysis
eTable 2. Inclusion and Exclusion Criteria for Each Trial
eTable 3. Characteristics of the Population
eFigure 1. Estimated Probability of 90-day Mortality Based on SAPS II and on the Optimal Individual Model
eFigure 2. Distribution of the Estimated Individual Treatment Effect by Steroid Regimen
eFigure 3. Distribution of Maximal Absolute Risk Difference by Treatment Strategy
eFigure 4. Net Benefit According to the Proportion of Patients Receiving Treatment
eFigure 5. Estimated Net Benefit Based Number Willing to Treat in the External Validation Cohort
eFigure 6. Decision Tree for a Number Willing to Treat of 50 Patients
eTable 4. Characteristics of the Patients With a Estimated Individual Treatment Effect Within the First vs Fourth Quartiles

This supplemental material has been provided by the authors to give readers additional information about their work.

eTable 1. Description of Studies Included in the Analysis

	GER-inf-05 [16]	CORTICUS [17]	COIITSS [18]	APROCCHSS [19]	Arabi et al. [20]
Design	Randomized, double-blind, parallel-group and placebo-controlled trials	Multicenter, randomized, double-blind, parallel-group and placebo-controlled trials	Multicenter, open-labelled, randomized, 2-by-2 factorial trial	Multicenter, double-blind, randomized, 2-by-2 factorial trial	Randomized double-blinded placebo-controlled trial
Inclusion dates	10/1995 – 02/1999	03/2002 – 11/2005	01/2006 – 01/2009	09/2008 - 06/2015	04/2004 – 10/2007
Publication date	2002	2008	2010	2018	2010
Sample size	300	499	509	1241	75
Groups					
Experimental	Hydrocortisone + Fludrocortisone	Hydrocortisone	Hydrocortisone + Fludrocortisone	Hydrocortisone + Fludrocortisone	Hydrocortisone
Control	Placebo	Placebo	Hydrocortisone	Placebo	Placebo
Corticosteroid regimen	7-day course of 200mg intravenous hydrocortisone plus 50µg enteral fludrocortisone	hydrocortisone at a daily dose of 200mg for 5 days then tapered off over 6 days	50-mg intravenous bolus of hydrocortisone every 6 hours for 7 days + 50 µg through the nasogastric tube for a similar duration	50 mg as intravenous bolus every 6 hours for 7 days + 50 µg through the nasogastric tube for a similar duration	50mg iv bolus every 6 hours until shock resolution, followed by a tapering off over 8 days
Other treatment tested	-	-	Tight glycemic control	activated drotrecogin alfa	-
Primary outcome	28-day mortality	28-day mortality	In-hospital or 90-day mortality whichever occurred first	90-day mortality	28-day mortality
Follow up	1 year	1 year	180 days	180 days	Hospital stay
Corticotropin stimulation test	Yes	Yes	Yes	Yes	Yes
Tapering	No	Yes	No	No	Yes

© 2020 Pirracchio R et al. *JAMA Network Open.*

eTable 2. Inclusion and Exclusion Criteria for Each Trial

	Inclusion	Exclusion
GER-inf-05	Clinical evidence of infection<br>SBP < 90 mmHg AND vasopressor<br>Urinary output < 0.5mL/kg<br>PaO2/FiO2 < 280mmHg<br>Arterial lactate > 2 mmol/L<br>Need for mechanical ventilation 8h after onset of shock	Pregnancy<br>Acute myocardial infarction, pulmonary embolism, advanced form of cancer or HIV<br>Contraindication or formal indication for corticosteroids<br>Etomidate within 6h prior to randomization
CORTICUS	Clinical evidence of infection<br>SBP < 90 mmHg OR vasopressor<br>Any hypoperfusion or organ dysfunction<br>72h after onset of shock	Underlying poor prognosis or immunosuppression<br>Prior corticosteroid medication<br>Life expectancy < 24h
COIITSS	Criteria for severe sepsis<br>SBP ≤ 90mmHg or MAP ≤ 60 mmHg AND vasopressor<br>SOFA score ≥ 8<br>3h after onset of shock	Pregnancy<br>Life expectancy < 24h
APROCCHSS	Indisputable or probable septic shock for less than 24 hours. Presence of a clinically or microbiologically documented infection, a SOFA score of 3 or 4 for at least two organs and at least 6 hours, and receipt of vasopressor therapy (norepinephrine, epinephrine, or any other vasopressor at a dose of ≥0.25 µg per kilogram of body weight per minute or ≥1 mg per hour) for at least 6 hours to maintain a systolic blood pressure of at least 90 mm Hg or a mean blood pressure of at least 65 mm Hg.	Presence of septic shock for at least 24 hours,<br><br>high risk of bleeding,<br><br>pregnancy or lactation,<br><br>underlying conditions that could affect short-term survival,<br><br>known hypersensitivity to drotrecogin alfa (activated), or previous treatment with corticosteroids. After the withdrawal of Xigris from the market, the exclusion criteria that were relevant only to drotrecogin alfa (activated) were removed.

© 2020 Pirracchio R et al. *JAMA Network Open.*

eTable 3. Characteristics of the Population

	Hydrocortisone + Fludrocortisone n = 1009	Hydrocortisone n = 515	Placebo n = 1024	Overall n = 2548
Age	66 [56, 76]	65 [53, 74]	67 [55, 76]	66 [55, 76]
SAPS2	57 [43, 70]	55 [41, 68]	53 [41, 67]	55 [42, 69]
SOFA day 1	11 [9, 13]	11 [8, 13]	11 [9, 13]	11 [9, 13]
Mechanical ventilation	959 (95%)	492 (96%)	931 (91%)	2382 (93%)
Arterial lactate (mmol/L)	2.8 [1.8, 4.9]	2.9 [1.8, 4.8]	2.9 [1.8, 5.1]	2.8 [1.8, 4.9]
Baseline cortisol (μg/dl)	119 [24, 328]	27 [17, 42]	97 [23, 311]	47 [21, 245]
ACTH responder	298 (29%)	207 (40%)	340 (33%)	845 (33%)
Etomidate	40 (4%)	56 (11%)	85 (8%)	181 (7%)
Blood sugar inclusion (mg/dl)	173 [130, 228]	151 [119, 203]	151 [116, 210]	160 [121, 216]
Origin of Infection				
Community-acquired	728 (72%)	303 (59%)	682 (67%)	1713 (67%)
Hospital-acquired	269 (27%)	211 (41%)	321 (31%)	801 (31%)
Reason for ICU admission				
Medical	580 (57%)	79 (15%)	681 (67%)	1340 (53%)
Elective surgery	23 (2%)	22 (4%)	53 (5%)	98 (4%)
Emergent surgery	148 (15%)	142 (27%)	274 (27%)	564 (22%)
Site of Infection				
Bacteremia	45	18	49	112
GI	91	128	163	382
Lung	422	137	399	958
Soft tissue	37	17	41	95
Urinary tract	62	22	81	165
Bones, joints	0	5	1	6
CNS	3	5	0	8
Endocarditis	3	3	2	8
Multiple	242	138	182	562
Others	81	35	85	201
Pathogen				
Only Gram positive	243	116	249	608
Only Gram negative	255	164	278	697
Only fungus	29	22	33	84
Only anaerobes	8	11	11	30
Gram positive and Gram negative	100	21	79	200
Other	30	16	11	57
Other mixed	35	0	36	71
Pathogen unknown	309	165	326	800
28-day mortality	380 (38%)	188 (37%)	413 (40%)	981 (39%)
90-day mortality	470 (47%)	231 (45%)	514 (50%)	1215 (48%)

Percentages not adding up to 100% are explained by missing data.

© 2020 Pirracchio R et al. *JAMA Network Open.*

eFigure 1. Estimated Probability of 90-day Mortality Based on SAPS II and the Optimal Individual Model

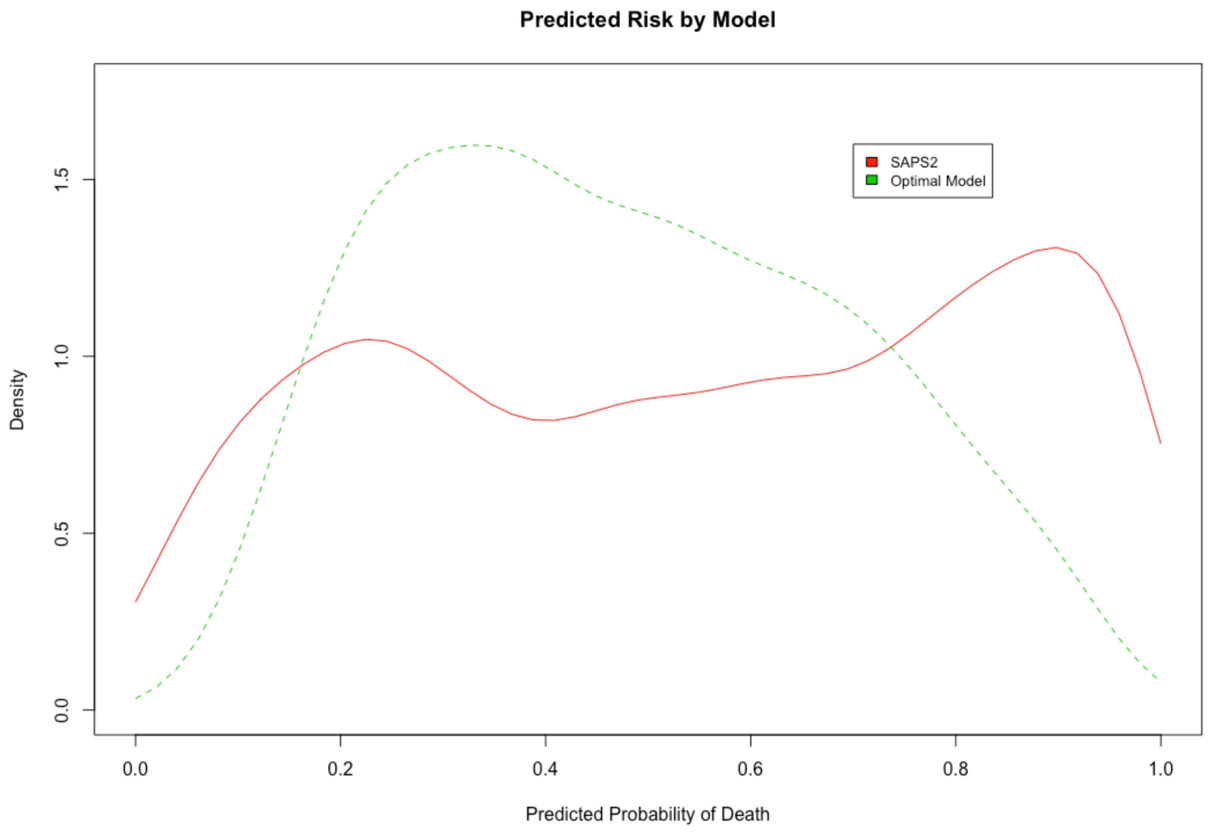

© 2020 Pirracchio R et al. *JAMA Network Open.*

eFigure 2. Distribution of the Estimated Individual Treatment Effect by Steroid Regimen
The ITE is expressed as an absolute risk reduction.

A. Severity of Illness Model

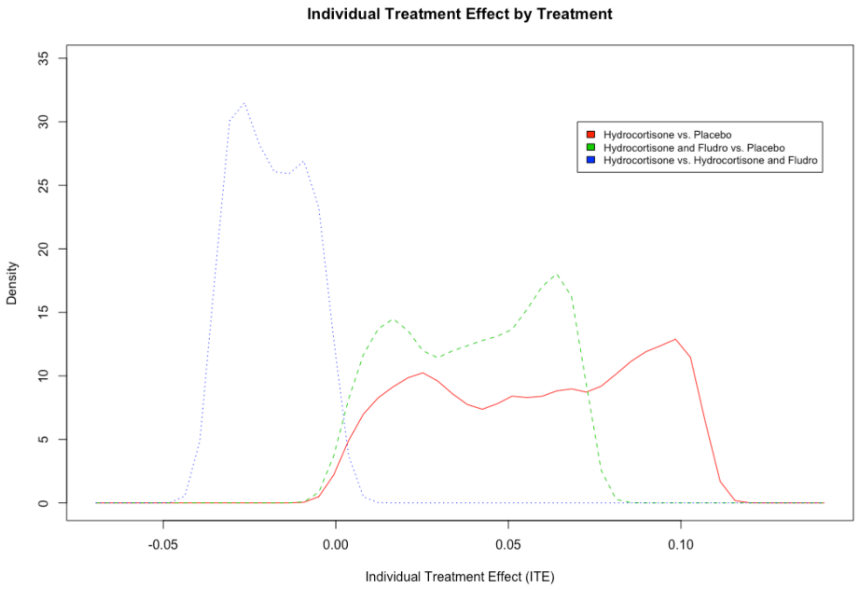

© 2020 Pirracchio R et al. *JAMA Network Open.*

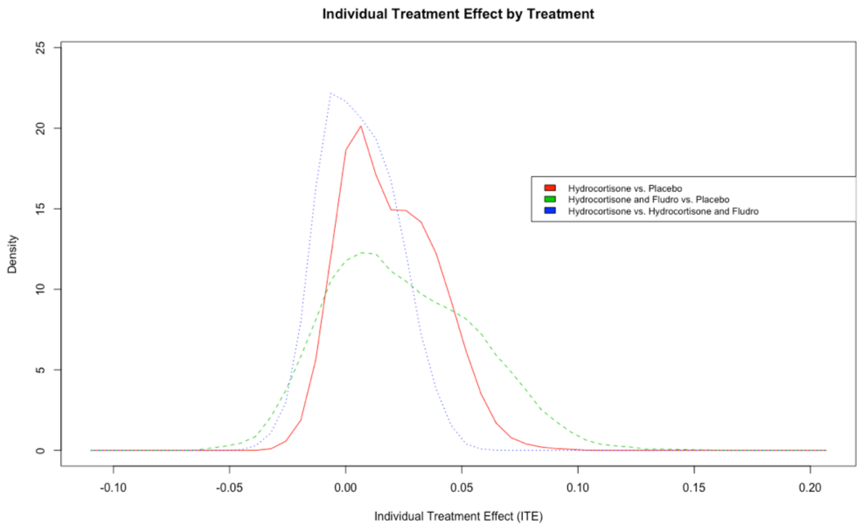

B. Optimal Individual Model

© 2020 Pirracchio R et al. *JAMA Network Open.*

eFigure 3. Distribution of Maximal Absolute Risk Difference[a] by Treatment Strategy

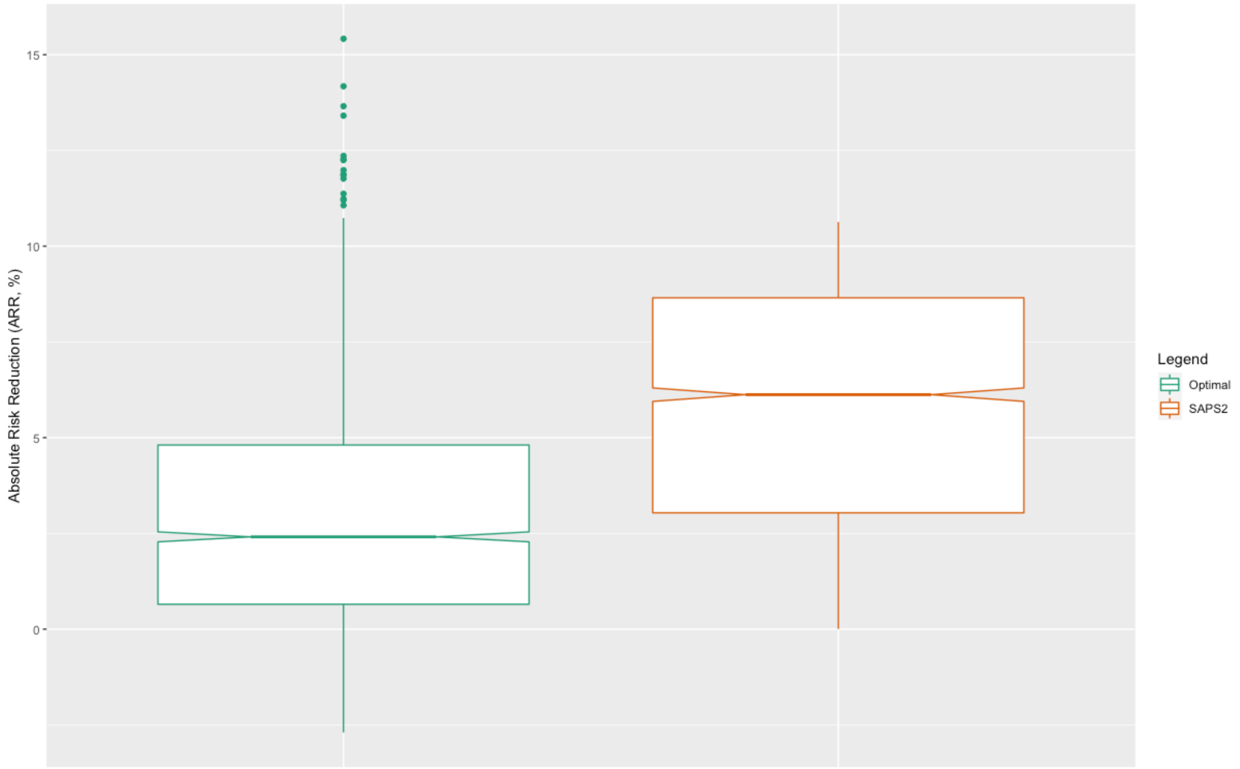

[a] The maximal absolute risk difference refers to the maximal individual treatment effect obtained with either hydrocortisone and fludrocortisone or with hydrocortisone alone.

© 2020 Pirracchio R et al. *JAMA Network Open.*

eFigure 4. Net Benefit According to the Proportion of Patients Receiving Treatment

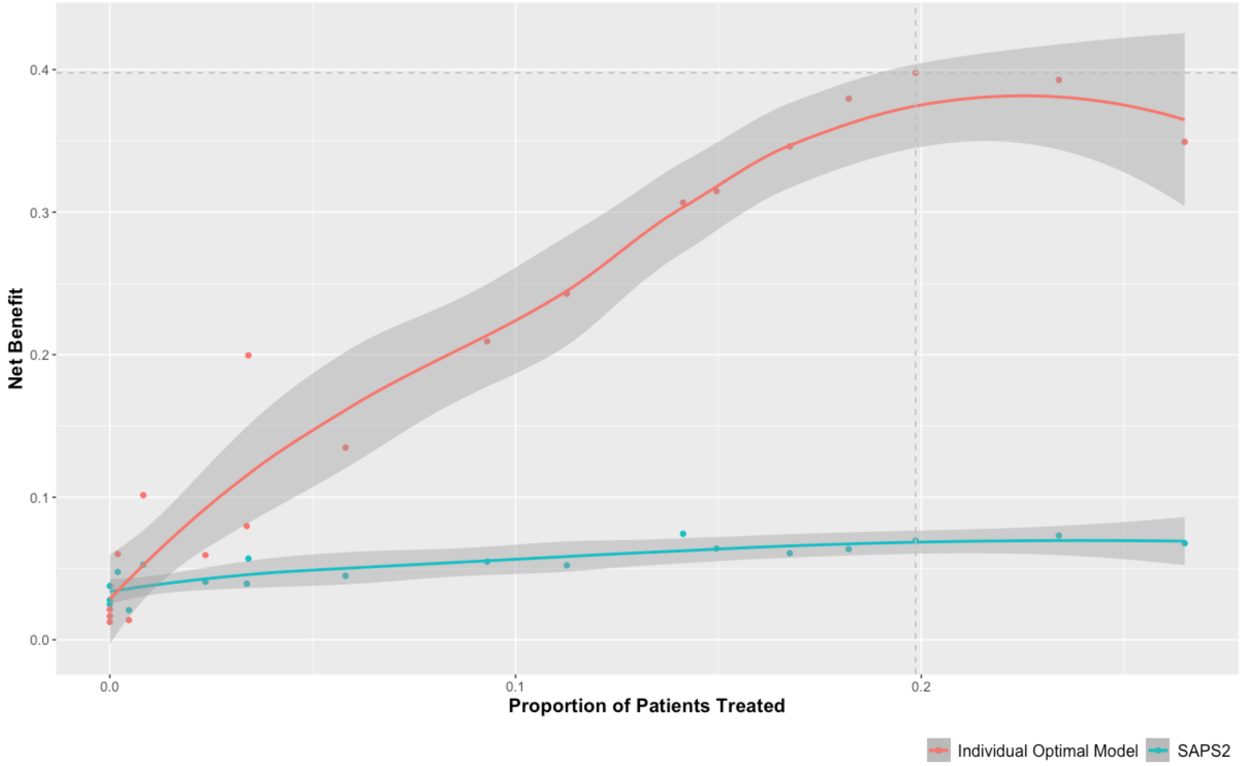

A maximal net benefit for the optimal individual treatment rule is obtained for a proportion of patients treated of 20%, which correspond to a treatment decision threshold of 2.5%, i.e. an absolute risk difference of at least 2.5% between treated and untreated.

© 2020 Pirracchio R et al. *JAMA Network Open.*

eFigure 5. Estimated Net Benefit Based Number Willing to Treat in the External Validation Cohort

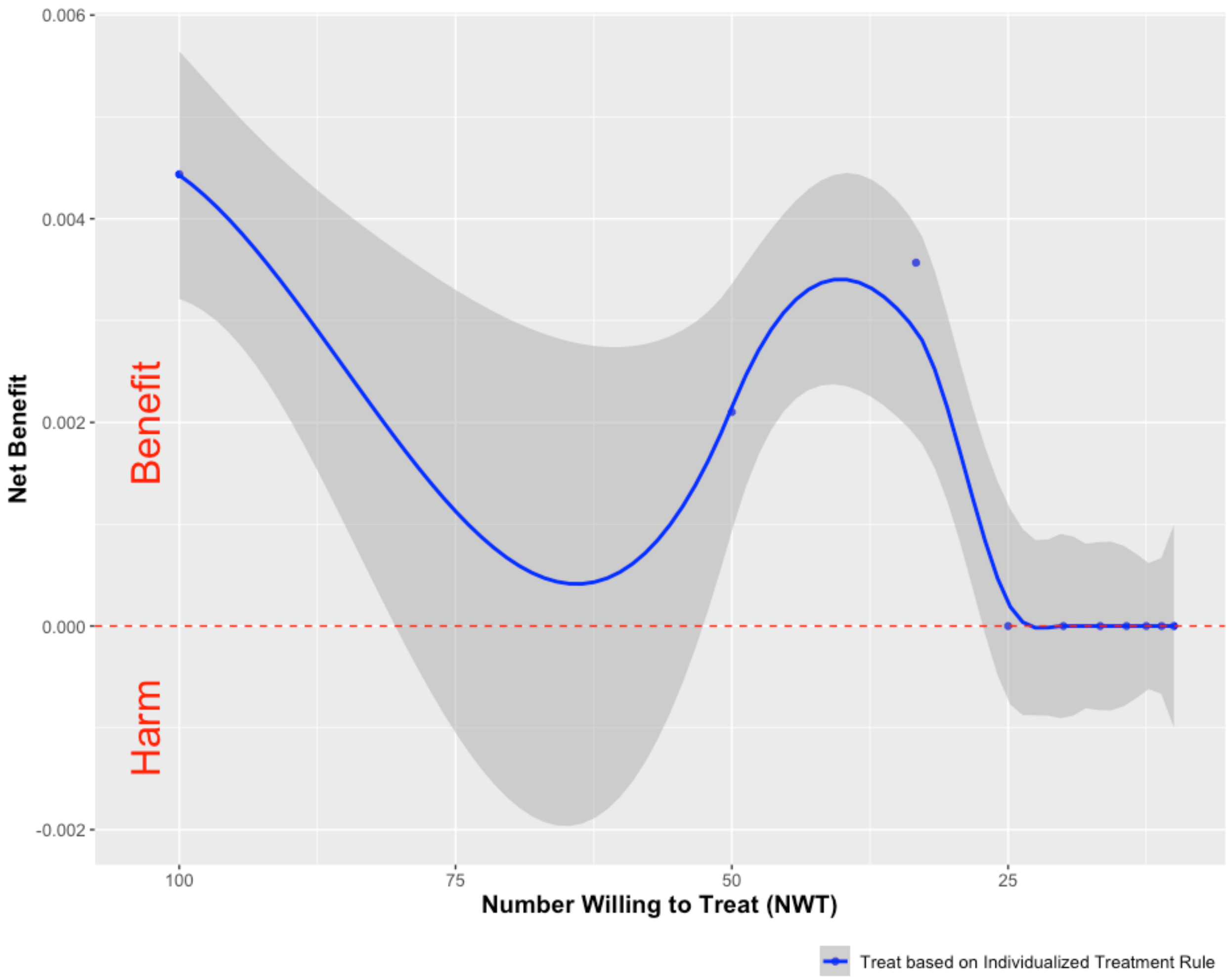

The y-axis is the net benefit for each treatment strategy compared to treating no one. Treating no one served as a reference and is equal to zero. The x-axis is the number willing to treat (NWT) which is equal to 1 / decision threshold.

© 2020 Pirracchio R et al. *JAMA Network Open.*

eFigure 6. Decision Tree for a Number Willing to Treat of 50 Patients

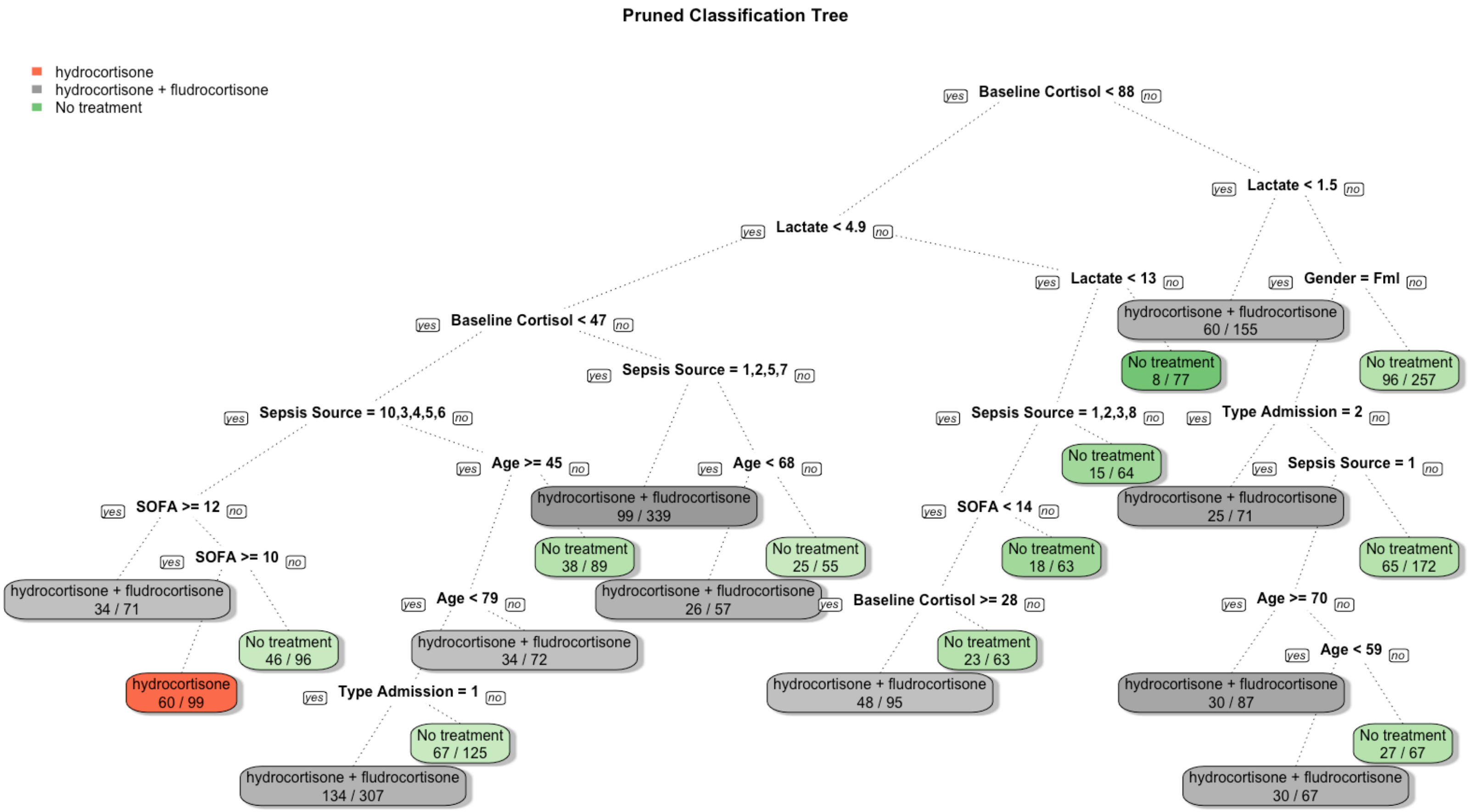

Sepsis source – 1: bacteriemia; 2: abdominal; 3: lung; 4: multiple; 5: other; 6: soft tissue; 7: urinary tract; 8: bones and joints; 9: central nervous system; 10: endocarditis
Type admission - 1 : medical ; 2 : elective surgery ; 3: emergency surgery
Fml: female

© 2020 Pirracchio R et al. *JAMA Network Open.*

eTable 4. Characteristics of the Patients With a Estimated Individual Treatment Effect Within the First and Fourth Quartiles

	Low ITE n = 637	High ITE n = 637
Age	65 [53, 77]	70 [61, 77]
SAPS2	55 [42, 72]	55 [42, 66]
SOFA day 1	11 [9, 14]	11 [9, 12]
Mechanical ventilation	542 (85%)	625 (98%)
Arterial lactate (mmol/L)	3.5 [2.1, 6.8]	2.3 [1.6, 3.7]
Baseline cortisol (µg/dl)	97 [25, 290]	48 [22, 257]
ACTH responder	215 (40%)	171 (40%)
Etomidate	38 (6%)	31 (5%)
Blood sugar inclusion (mg/dl)	160 [112, 218]	165 [130, 221]
Origin of Infection		
Community-acquired	408 (64%)	478 (75%)
Hospital-acquired	216 (40%)	154 (24%)
Reason for ICU admission		
Medical	388 (61%)	365 (57%)
Elective surgery	40 (6%)	8 (1%)
Emergent surgery	117 (18%)	125 (20%)
Site of Infection		
Bacteremia	46	13
GI	95	69
Lung	199	302
Soft tissue	36	16
Urinary tract	43	37
Bones, joints	1	1
CNS	1	0
Endocarditis	5	0
Multiple	120	163
Others	78	22
Pathogen		
Only Gram positive	175	144
Only Gram negative	189	147
Only fungus	19	25
Only anaerobes	10	4
Gram positive and Gram negative	46	63
Other	6	25
Other mixed	23	12
Pathogen unknown	168	217
28-day mortality	266 (42%)	201 (32%)
90-day mortality	310 (52%)	277 (45%)

© 2020 Pirracchio R et al. *JAMA Network Open.*
